# Exatecan Antibody Drug Conjugates Based on a Hydrophilic Polysarcosine Drug-Linker Platform

**DOI:** 10.3390/ph14030247

**Published:** 2021-03-09

**Authors:** Louise Conilh, Guy Fournet, Eric Fourmaux, Angélique Murcia, Eva-Laure Matera, Benoît Joseph, Charles Dumontet, Warren Viricel

**Affiliations:** 1 Mablink Bioscience, 69 rue de la république, 69002 Lyon, France; l.conilh@mablink.com (L.C.); e.fourmaux@mablink.com (E.F.); a.murcia@mablink.fr (A.M.); 2 Centre de Recherche en Cancérologie de Lyon, INSERM 1052, CNRS 5286, Université de Lyon, 69008 Lyon, France; eva-laure.matera@univ-lyon1.fr (E.-L.M.); charles.dumontet@chu-lyon.fr (C.D.); 3 Institut de Chimie et Biochimie Moléculaires et Supramoléculaires, UMR CNRS 5246, Université de Lyon, 69100 Villeurbanne, France; guy.fournet@univ-lyon1.fr (G.F.); benoit.joseph@univ-lyon1.fr (B.J.); 4 Hospices Civils de Lyon, 69000 Lyon, France

**Keywords:** antibody–drug conjugates, polysarcosine, deruxtecan, topoisomerase I inhibitor, camptothecin

## Abstract

We herein report the development and evaluation of a novel HER2-targeting antibody–drug conjugate (ADC) based on the topoisomerase I inhibitor payload exatecan, using our hydrophilic monodisperse polysarcosine (PSAR) drug-linker platform (PSARlink). In vitro and in vivo experiments were conducted in breast and gastric cancer models to characterize this original ADC and gain insight about the drug-linker structure–activity relationship. The inclusion of the PSAR hydrophobicity masking entity efficiently reduced the overall hydrophobicity of the conjugate and yielded an ADC sharing the same pharmacokinetic profile as the unconjugated antibody despite the high drug-load of the camptothecin-derived payload (drug–antibody ratio of 8). Tra-Exa-PSAR10 demonstrated strong anti-tumor activity at 1 mg/kg in an NCI-N87 xenograft model, outperforming the FDA-approved ADC DS-8201a (Enhertu), while being well tolerated in mice at a dose of 100 mg/kg. In vitro experiments showed that this exatecan-based ADC demonstrated higher bystander killing effect than DS-8201a and overcame resistance to T-DM1 (Kadcyla) in preclinical HER2+ breast and esophageal models, suggesting potential activity in heterogeneous and resistant tumors. In summary, the polysarcosine-based hydrophobicity masking approach allowsfor the generation of highly conjugated exatecan-based ADCs having excellent physicochemical properties, an improved pharmacokinetic profile, and potent in vivo anti-tumor activity.

## 1. Introduction

Antibody-drug conjugates (ADCs) are biotherapeutics that combine highly cytotoxic molecules with the targeting property of antibodies to specifically kill cancer cells [1]. These agents possess reduced systemic toxicity and aim to improve the narrow therapeutic window associated with conventional chemotherapies. One hundred years after the emergence of the Magic Bullet concept [2], 10 ADCs are now clinically approved, 5 of which were approved during the past two years [3,4,5,6,7]. More than 90 ADCs are currently under clinical evaluation [8,9]. The vast majority of approved ADCs as well as those currently in clinical trials deliver microtubule inhibitors (auristatins, maytansinoids) or DNA-alkylating agents (calicheamicin, pyrrolobenzodiazepines, duocarmycins) and are limited to a drug-antibody ratio (DAR) of 2 to 4. This moderate DAR value has until recently been considered to be optimal to obtain efficient ADCs achieving acceptable pharmacokinetic properties, in vivo efficacy, and safety. This is due to intrinsic hydrophobicity of the drug-linker, which negatively impacts antibody tertiary structure, causes increased plasma clearance, increases toxicity, and ultimately leads to a reduced therapeutic window [10,11,12,13,14]. In this context and because only 1–2% of the injected payload dose ultimately reach the tumor site [15], highly cytotoxic small molecules with IC_50_’s in the pico- to low nano-molar range are therefore required to generate moderately conjugated yet potent ADCs [16,17]. This finding has greatly oriented the field towards ADCs based on very potent DNA alkylating agents (such as pyrrolobenzodiazepines) and site-specific DAR 2 bioconjugation technologies [18,19,20]. Several promising and strongly cytotoxic ADCs entered clinical trials but showed unsatisfactory results due to non-manageable undesirable off-target toxicities [21,22], suggesting that ADCs based on less potent but safer cytotoxic small molecules could benefit from a wider clinical therapeutic window.

Topoisomerase I (Topo I) inhibitors represent the most recent breakthrough in ADC payload innovation with the approval of two ADCs containing camptothecin (CPT) analogues: trastuzumab deruxtecan (DS-8201a—Enhertu^®^) and sacituzumab govitecan (IMMU-132—Trodelvy^TM^) [23,24]. Topo I inhibitors trigger cell apoptosis through their specific binding at the DNA–topoisomerase interface, leading to the inhibition of DNA supercoiling and entanglement, resulting in DNA damage and cell death [25,26]. This class of payloads is mainly composed of CPT analogues, wherein irinotecan and topotecan are FDA-approved chemotherapeutics [27,28]. Topo I inhibitors are 10- to 100-fold less potent than microtubule-targeting and DNA-alkylating agents, which largely explains the lack of initial interest for these payloads in first generation ADCs. However the recent development of carefully designed hydrophilic drug-linkers able to overcome payload hydrophobicity in order to generate highly-conjugated ADCs [29,30,31,32,33,34,35] allows us to reconsider Topo I inhibitors as potential ADC payloads. This is illustrated by the recent FDA approval of trastuzumab deruxtecan for the treatment of unresectable or metastatic HER2+ breast cancer and later in 2021 for the treatment of HER2+ locally advanced or metastatic gastric cancer. This ADC is composed of the HER2-targeting monoclonal antibody (mAb) trastuzumab, homogeneously attached to eight molecules of deruxtecan, a drug-linker based on the active topo I inhibitor agent DXd and a novel GGFG quadripeptide-based cleavable moiety [23]. Preclinical evaluations and clinical trials demonstrated a favorable pharmacokinetic profile, excellent anti-tumor activity and safety profiles, as well as a strong bystander killing effect, surpassing the previously approved trastuzumab emtansine (T-DM1—Kadcyla^®^) in several studies [23,36,37,38,39,40,41].

We have previously reported a hydrophilic monodisperse polysarcosine-based drug-linker platform (PSARlink^TM^) that demonstrated significant reduction in ADC hydrophobicity level in spite of high DAR 8 MMAE conjugation, associated with improved physicochemical and pharmacological properties [29]. In the present work, we report the translation of our ADC platform to the topoisomerase I inhibitor compound exatecan. This active agent presents promising potential but its use as an ADC payload has been limited because of its hydrophobicity and challenging biophysical properties, which appear to be strongly caused by the steric hindrance around the stereo-defined primary amine [42]. Exatecan is a partially water-soluble and non-prodrug derivative of CPT [43] that is closely related but not identical to DXd (Figure S1). The conjugation of exatecan to trastuzumab through the glucuronidase-cleavable polysarcosine-based linker reported therein (Exa-PSAR10) ultimately provides (i) a homogeneous DAR 8 conjugation, (ii) an improved hydrophilic profile of the resulting ADC, (iii) an exquisite drug-linker plasma stability and favorable pharmacokinetic profile, (iv) a potent in vivo activity against breast and gastric cancer models, and (v) a highly potent in vitro bystander killing effect as well as a strong cytotoxicity against T-DM1-resistant cells.

## 2. Results

### 2.1. Drug-Linker Conception, Bioconjugation, and Physicochemical Characterization of ADCs

**Figure 1:**
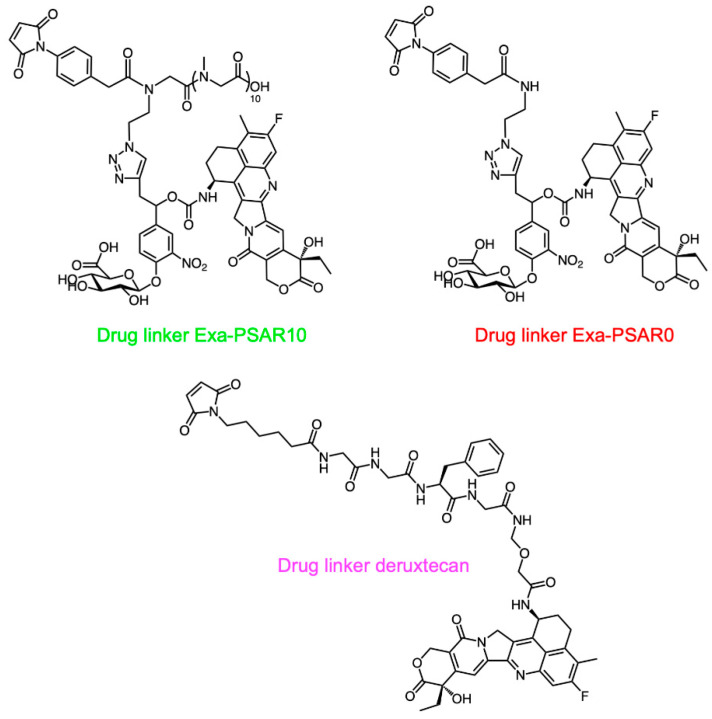
Chemical structures of the antibody-drug conjugates (ADC) drug-linkers used in the present study. See Supplementary Materials for detailed chemical synthetic procedures and exact ADC structures.

Following known chemical procedures [29], synthesis of the Exa-PSAR10 drug-linker was conducted (Figure 1 and Supplementary Materials). This enzyme-cleavable drug-linker includes a β-glucuronidase-sensitive trigger unit [44] to allow intracellular release of the final cytotoxic agent exatecan after endocytosis of the ADC [45]. To maximize drug-linker hydrophilicity, we included an orthogonal monodisperse PSAR unit as an hydrophobicity masking entity [29]. As we knew that a PSAR length of 12 sarcosine residues was an optimal value for the monomethyl auristatin E (MMAE) payload [29] and that exatecan is slightly less hydrophobic than MMAE (logP_exatecan_ = 1.67–3.29 and logP_MMAE_ = 3.44–4.61 as predicted in silico [46]), we selected a length of 10 sarcosine residues. Finally, an auto-hydrolyzable aryl-maleimide bioconjugation head [47] was selected to prevent premature and deleterious in vivo deconjugation of the drug-linker by retro-Michael rearrangement with albumin in plasma [48]. A drug-linker Exa-PSAR0 lacking the PSAR hydrophobicity masking unit was also synthesized and was used as a negative control.

Drug-linkers were conjugated to the native inter-chain cysteines of reduced trastuzumab following a straightforward bioconjugation protocol in order to obtain homogeneous DAR 8 ADCs (Figure S2). Bioconjugation yields were above 80% for Tra-Exa-PSAR10 and Tra-Exa-PSAR0 ADCs, and around 60–70% for Tra-deruxtecan (DS-8201a linker). Analytical characterization by denaturing reversed phase chromatography–mass spectrometry (RPLC–MS) confirmed efficient conjugation and homogeneity of the conjugates (Figure S3).

**Figure 2:**
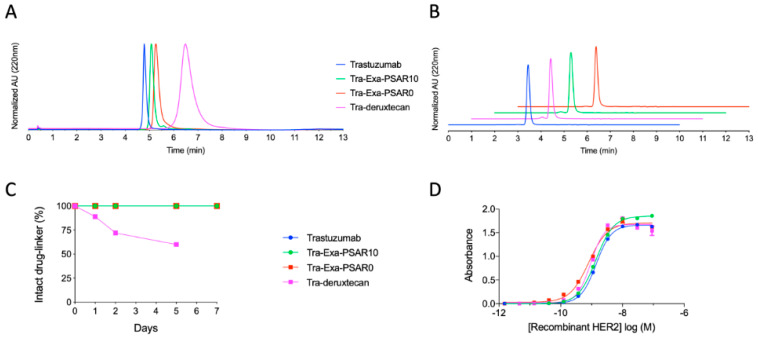
Physicochemical characterization of ADCs. (**A**) Hydrophobic Interaction Chromatograms (HIC) of DAR8 ADCs. (**B**) Size Exclusion Chromatograms (SEC) of DAR8 ADCs. (**C**) Ex-vivo rat plasma stability studies, as assayed by immunocapture and reversed phase HPLC—mass spectrometry. Low immunocapture recovery of the heavy-chain of the Tra-deruxtecan conjugate prevented us to report stability data at day 7 for this ADC. (**D**) HER2 ELISA binding affinity profiles of ADCs.

Hydrophobic interaction chromatography (HIC) profiles of the conjugates showed that the two ADCs based on the glucuronide-exatecan modality (Tra-Exa-PSAR10 and Tra-Exa-PSAR0) appeared almost as hydrophilic as the native antibody, despite the grafting of eight exatecan payloads per antibody (Figure 2A). No significant differences in HIC retention time were observed between the PSAR10 ADC and the PSAR0 negative control ADC. This result was not in accordance with our previous work with MMAE payload [29], where the negative control ADC lacking the hydrophobicity masking entity (PSAR0) showed a very significant increase in HIC retention time. The Tra-deruxtecan ADC eluted later in the HIC chromatogram, indicating a higher hydrophobicity level compared to the Exa-PSAR10 and Exa-PSAR0 conjugates. Size exclusion chromatography (SEC) chromatograms indicated that all ADCs were 95%+ monomeric and that no aggregation occurred during formulation (Figure 2B). Ex vivo rat plasma stability studies by immunocapture followed by RPLC–MS were also conducted (Figure 2C). Tra-Exa-PSAR10 and Tra-Exa-PSAR0 were found to be stable during the 7-day incubation period, as no premature glucuronide-cleavage was observed as well as no maleimide deconjugation. This could be expected as these two drug-linkers incorporate a stabilized auto-hydrolyzable maleimide bioconjugation head [47]. It was observed that Tra-deruxtecan lost approximately 20–40% of its cargo by maleimide deconjugation during the first days of incubation. This result is expected as the maleimidocaproyl bioconjugation headgroup that is used in the deruxtecan and vedotin (mc-vc-PAB-MMAE) drug-linkers is known to be susceptible to such retro-Michael deconjugations [48,49].

### 2.2. DAR8 Conjugation and Orthogonal Polysarcosine Inclusion Did Not Negatively Impact Binding to HER2

**Figure 3:**
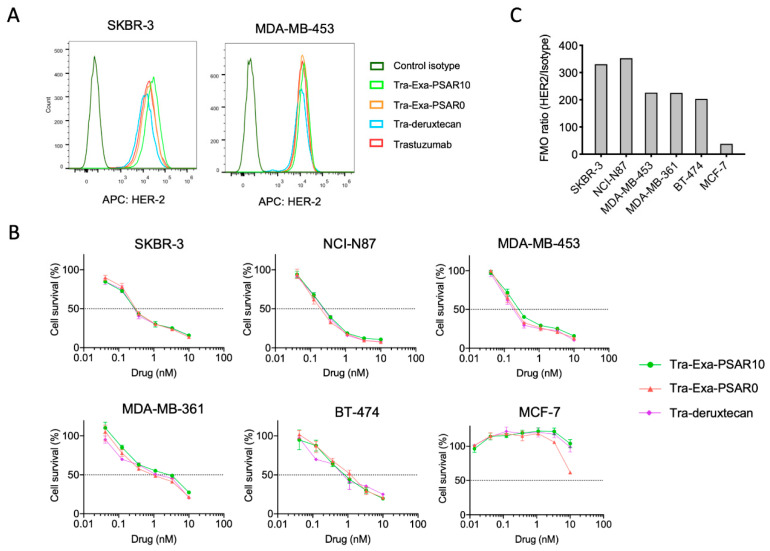
In vitro evaluation of Tra-Exa-PSAR10. (**A**) Trastuzumab and ADC cell binding assayed by flow cytometry. (**B**) In vitro cytotoxicity of ADCs in breast and gastric HER2+ and HER2- (MCF-7) cancer cell lines after 6-day exposure to trastuzumab conjugates, as assayed by MTT assay, *n* = 3. (**C**) HER2 cell surface expression in breast and gastric tumor cell lines as assayed by flow cytometry.

Antigen binding of unconjugated trastuzumab and trastuzumab conjugated either to Exa-PSAR10, Exa-PSAR0, or deruxtecan drug-linkers was assessed by ELISA (Figure 2D). No antigen-binding alteration of trastuzumab was observed when the antibody was fully reduced and conjugated at DAR 8 with each of the three drug-linkers, as log(EC50) values were −8.9, −8.9, −9.0, and −9.1. In vitro cell binding was also assessed by flow cytometry using APC-labelled versions of the unconjugated and conjugated trastuzumab in HER-2-positive cell lines (Figure 3A). Similar level of binding of the four tested entities was observed in two HER2-positive cell lines, SKBR-3 and MDA-MB-453, as compared to the isotype control. These results suggest that neither DAR 8 conjugation nor orthogonal incorporation of the polysarcosine hydrophobicity masking entity negatively impacted the antibody quaternary structure or masked its complementarity-determining regions (CDR).

### 2.3. Tra-Exa-PSAR10 ADC Displayed Strong In Vitro Cytotoxicity in HER2-Positive Cell Lines

In vitro activity of Tra-Exa-PSAR10 was assessed by in vitro cytotoxicity assay and compared to Tra-Exa-PSAR0 and Tra-deruxtecan (Figure 3B). The three ADCs presented equal sub-nanomolar IC_50_’s in the five HER2-expressing cell lines (Figure 3C), i.e., SKBR-3 (IC_50_ = 0.18 ± 0.04 nM), NCI-N87 (IC_50_ = 0.20 ± 0.05 nM), MDA-MB-453 (IC_50_ = 0.20 ± 0.10 nM), MDA-MB-361 (IC_50_ = 2.0 ± 0.8 nM), and BT-474 (IC_50_ = 0.9 ± 0.4 nM), and no cytotoxicity against MCF-7 (IC_50_ > 10 nM), a HER-2 negative breast cancer cell line. These results are in accordance with previously reported in vitro cytotoxicity data with DS-8201a on SKBR-3 and NCI-N87 cell lines with IC_50_s of 0.05 and 0.17 nM, respectively [23]. This series of experiments suggest that highly conjugated exatecan-based ADCs display strong in vitro cytotoxicity against breast and gastric cancer cells, comparable to that of the FDA-approved ADC trastuzumab-deruxtecan (DS-8201a).

### 2.4. Tra-Exa-PSAR10 Showed a Favorable PK Profile and Strong In Vivo Activity in HER2+Breast and Gastric Cancer Models

**Figure 4:**
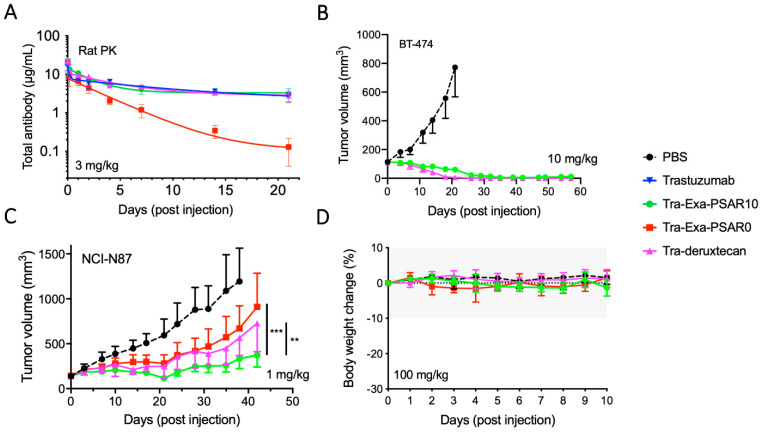
In vivo evaluation of Tra-Exa-PSAR10. (**A**) ADC pharmacokinetic study in Sprague-Dawley rats after a single intravenous ADC dose of 3 mg/kg. Total ADC concentration was assayed by anti-human IgG ELISA. (**B**) Antitumor activity in HER2+ SCID/BT-474 breast cancer model following a single intravenous ADC dose of 10 mg/kg. (**C**) Antitumor activity in HER2+ SCID/NCI-N87 gastric cancer model following a single sub-curative intravenous ADC dose of 1 mg/kg. (**D**) Mice tolerability experiment following a single intraperitoneal ADC dose of 100 mg/kg. (**: *p* < 0.01, ***: *p* < 0.001).

We first assessed the total ADC pharmacokinetic (PK) profile of Tra-Exa-PSAR10 in Sprague-Dawley rats using anti-human IgG ELISA, and compared it to unconjugated trastuzumab, Tra-Exa-PSAR0, and Tra-deruxtecan. A dose of 3mg/kg was selected on the basis of previous knowledge with DAR8 ADCs [29,30] and to be above saturable dose range in order to obtain linear and predictable clearance profiles [50]. As previously observed with highly conjugated antibodies [13,29], trastuzumab conjugated to eight molecules of exatecan (Tra-Exa-PSAR0) and exhibited an unfavorable accelerated plasma clearance (Figure 4A and Figure S4). The excessive hydrophobicity of highly conjugated ADCs has been shown to be the major factor of a poor pharmacokinetic profile [12]. The orthogonal inclusion of the PSAR10 hydrophobicity masking entity to the drug-linker structure successfully restored the same PK profile as the native unconjugated trastuzumab. Interestingly, the PK profile of Tra-deruxtecan also mimicked that of trastuzumab, despite the fact that this ADC was found to be somewhat hydrophobic by HIC (Figure 2A).

As a first preliminary in vivo efficacy experiment, Tra-Exa-PSAR10 and Tra-deruxtecan were evaluated in the HER2+ breast cancer model BT-474 (Figure 4B). At a single exploratory ADC dose of 10 mg/kg, a complete and prolonged remission of the tumors was observed in all groups. To further explore potential discrepancies in activity between the three drug-linkers of the study, we used the HER2+ gastric cancer model NCI-N87 at a sub-curative ADC dose of 1 mg/kg (Figure 4C). This dose was selected considering a previously reported NCI-N87 xenograft study with DS-8201a [37]. The tumor growth rate was significantly reduced in all groups compared to the control group, albeit to different extents. The most potent anti-tumor activity was observed with Tra-Exa-PSAR10, which was significantly more efficacious than its PSAR-lacking counterpart Tra-Exa-PSAR0 and the DXd-based ADC Tra-deruxtecan (Figure S5). These results suggest that the favorable PK profile enabled by the presence of the PSAR hydrophobicity masking entity translates into an improved in vivo efficacy. A first exploratory tolerability assessment was then conducted in SCID mice for these three ADCs (Figure 4D). No body weight changes, signs of distress, or inability to drink or eat were observed in animals for up to 10 days after an intraperitoneal injection of the ADCs at 100 mg/kg. These results suggest a good systemic tolerance for the three topoisomerase I inhibitor-based ADCs.

### 2.5. Tra-Exa-PSAR10 Displayed a Strong Bystander Activity In Vitro

**Figure 5:**
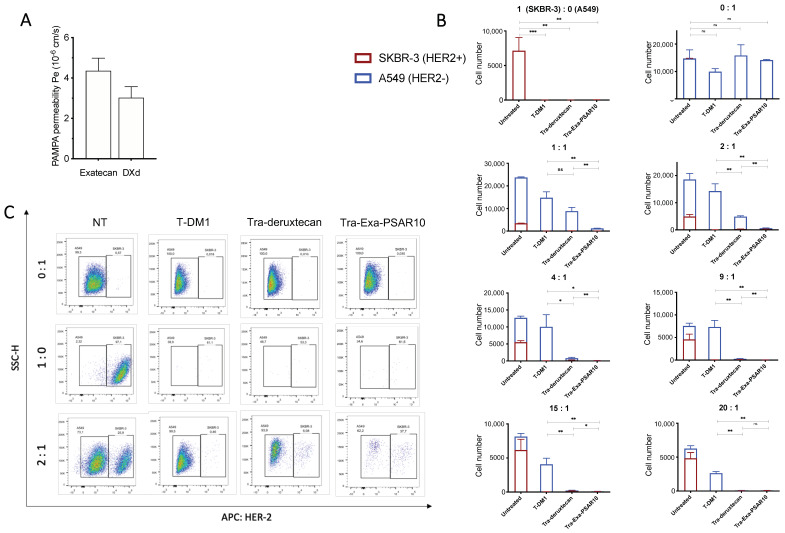
Bystander killing effect of Tra-Exa-PSAR10 compared to T-DM1 and trastuzumab-deruxtecan (DS-8201a) in co-culture in vitro. (**A**) Passive cell membrane diffusion of Exatecan and DXd payloads as assayed by a Corning^®^ Gentest^TM^ PAMPA assay. (**B**) In vitro bystander killing effect of Tra-Exa-PSAR10 compared to Tra-deruxtecan in co-cultured SKBR-3 (HER2+) and A549 (HER2-) cells that were treated with 10 nM ADCs for 5 days. Cell number and ratio of HER2+ and HER2- cells were determined by flow cytometry. T-DM1 (Kadcyla^®^) was used as a negative control. (**C**) Representative flow cytometry data presented for 0:1, 1:0 and 2:1 cell ratio, showing no impact of the ADCs on HER2- A549 cells (0:1), activity against HER2+ SKBR-3 cells (1:0) and a stronger bystander activity of Tra-Exa-PSAR10 compared to Tra-deruxtecan. ns: not significant, *: *p* < 0.05, **: *p* < 0.01, ***: *p* < 0.001). *n* = 3.

We first assessed the passive membrane diffusion of exatecan and compared its permeability coefficient (Pe) to DXd’s using a parallel artificial membrane permeability (PAMPA) assay (Figure 5A). Exatecan exhibited a higher Pe value compared to DXd (4.2 × 10^−6^ and 3.0 × 10^−6^ cm/s, respectively) suggesting a better ability to passively cross lipid cell membranes. We then investigated the bystander killing potential of Tra-Exa-PSAR10 using an in vitro culture system. Single cell lines and co-cultures of HER2- (A549) and HER2+ (SKBR-3) cell lines were performed at different ratios and exposed to either 10 nM of T-DM1 (negative control), DS-8201a, or Tra-Exa-PSAR10. After five days of incubation, cells were sorted for HER2 expression by flow cytometry. As expected, monocultured A549 cells displayed very modest to no sensitivity to the three ADCs at 10 nM, whereas the monocultured SKBR-3 cells were highly sensitive (Figure 5B,C). Co-culture experiments using various ratios of A549/SKBR3 cells showed that the HER2- A549 cells were killed when the co-cultures were exposed to DS-8201a and Tra-Exa-PSAR10 but not when exposed to T-DM1. Remarkably this effect was more pronounced for Tra-Exa-PSAR10 than for DS-8201a, especially at the 1:1; 1:2, and 1:4 cell ratios (HER2-A549/HER2+SKBR-3). This observation could be explained by the stronger passive membrane permeability of exatecan compared to that of DXd.

### 2.6. Tra-Exa-PSAR10 Overcame T-DM1 Resistance in Breast Cancer Models In Vitro

**Figure 6:**
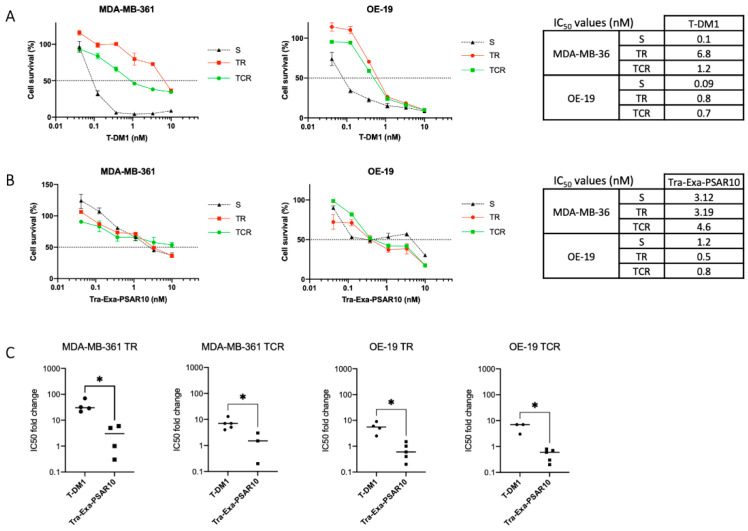
In vitro cytotoxicity of Tra-Exa-PSAR10 in cells resistant to T-DM1. (**A**) Cytotoxicity assay of T-DM1 in MDA-MB-361 and OE-19 sensitive (S) and T-DM1-resistant (TR and TCR) cells showing an increase in the IC_50_ values of resistant cells compared to parental. (**B**) Exposure of cells resistant to T-DM1 to Tra-Exa-PSAR10 showing comparable cytotoxicity in parental and T-DM1 resistant cells. (**C**) Relative resistance of TR and TCR cell lines to T-DM1 or Tra-Exa-PSAR10 represented as the IC_50_ of resistant cell line over the parental cell line for each experiment. *: *p* < 0,05. S: sensitive cells, TR: T-DM1 resistant cells and TCR: T-DM1 resistant cells generated in the presence of ciclosporin A.

T-DM1 (trastuzumab emtansine—Kadcyla^®^)-resistant cell lines were generated in our laboratory in HER2+esophageal OE-19 and HER2+ breast MDA-MB-361 cell lines, either in the absence (TR) or presence of ciclosporin A (TCR) [51,52]. This phenotype was confirmed using an in vitro cytotoxicity assay (Figure 6A). In MDA-MB-361 TR and TCR cell lines, IC_50_’s of T-DM1 were increased 21- and 7-fold, respectively, compared to the parental cell line. In OE-19 TR and TCR cell lines, IC_50_’s of T-DM1 were significantly increased sixfold compared to the parental OE-19-sensitive cell line.

Subsequently, the cytotoxicity of Tra-Exa-PSAR10 ADC was evaluated in these cell lines (Figure 6B,C). When exposed to Tra-Exa-PSAR10, the IC_50_ values of sensitive, TR, and TCR cells were, respectively, 3.1 nM, 3.2 nM, and 4.6 nM in the MDA-MB-361 models and 1.2 nM, 0.5 nM, and 0.8 nM in the OE-19 models. In accordance with previous studies [33,37], these results show that resistance mechanism to T-DM1 can be circumvented and that a comparable level of sensitivity between parental cells and T-DM1-resistant cells can be obtained with an ADC on the basis of a payload having a different mechanism of action.

## 3. Discussion

Most of the ADCs currently approved or in clinical trials are conjugated to microtubule inhibitors or DNA-alkylating agents, with a low-to-moderate DAR value. The recent approval of two DAR 8 ADCs containing topoisomerase I inhibitors (DS-8201a and IMMU-132) has enhanced the interest in the development of ADCs conjugated with this family of cytotoxic agents, which are 10- to 100-fold relatively less potent than the aforementioned payloads. One of the main challenges in the design of ADCs based on moderately potent payloads is thus to increase the DAR without disturbing antibody’s biophysical properties. The design of DS-8201a was based on a novel DXd payload and an optimized linker technology that integrates a quadripeptide cleavable unit coupled with a self-immolative amino methylene spacer moiety, allowing the DAR value to be increased to 8 [23]. In this context we produced a DAR 8 ADC based on exatecan, an alternative potent topoisomerase inhibitor, using a monodisperse polysarcosine-based hydrophobicity masking drug-linker technology, which we previously applied to MMAE [29]. This drug-linker has been found to be stable in rat plasma and displays a stronger bystander killing potential when compared to DS-8201a in vitro.

The physicochemical and in vitro characterization of Tra-Exa-PSAR10 compared to naked trastuzumab, the negative control Tra-Exa-PSAR0 lacking polysarcosine, and DS-8201a confirmed that neither the DAR 8 conjugation nor the incorporation of the PSAR entity affected the HER2-binding properties or the cytotoxicity of the ADCs. These data suggest that the size and the orthogonal attachment of the hydrophobicity masking entity does not negatively affect antibody binding, cell internalization, intracellular trafficking, endosomal linker cleavage, and active metabolite release. Exatecan- and DXd-based ADCs exhibited a potent and identical cytotoxicity in the low nanomolar range against breast and gastric cancer cell lines, both conjugates being compared at their identical DAR value of 8.

Numerous studies have demonstrated that increasing the DAR value causes an increase in the overall hydrophobicity of the ADC, which induces formation of aggregates, accelerated plasma clearance, off-target toxicity, and reduced anti-tumor activity [10,12,13,14,35]. The recent development of hydrophobicity masking-based ADC drug-linkers, such as PEG-incorporating linkers or polymeric polyacetal-based linkers, have demonstrated an efficient reduction of ADC overall hydrophobicity [14,29,30,31,32,34,53,54]. These approaches yielded maintained PK profiles and strong anti-tumor activities despite high DAR values (DAR 8-15) [31]. In our study, the PSAR incorporation in the drug-linker allowed us to generate a homogeneous DAR 8 ADC exhibiting a close-to-native-antibody hydrophobicity level as observed in hydrophobic interaction chromatography (HIC). The negative control ADC lacking PSAR surprisingly appeared almost as hydrophilic with this method, whereas DS-8201a showed a noticeable shift in retention time, indicating a higher hydrophobicity level. In vivo, all these ADCs exhibited a low clearance rate PK profile mimicking that of the native protein, except for the Tra-Exa-PSAR0 ADC. We observed that despite an apparently favorable HIC hydrophobicity level, this negative control ADC suffered from accelerated plasma clearance. The sterically hindered primary amine position of exatecan could be held responsible for this arguably unexpected result, as it has been reported that the lack of spacing at this critical position strongly influenced aggregation rates of the resulting ADCs [42]. This result suggests that designing an ADC drug-linker on the basis of an exatecan payload is not straightforward, and that simply combining exatecan with one of the most hydrophilic cleavable drug-linker unit that has been reported to date (β-glucuronide-based drug-linker) does not seem sufficient to ensure satisfactory in vivo properties.

DS-8201a showed a favorable rat PK profile despite its relatively high overall hydrophobicity level. This observation is in accordance with previously reported primate PK studies of this ADC, even though a comparison with the native antibody was not provided [39,55]. A possible explanation could be that the PK profile of DS-8201a is favored by the progressive deruxtecan drug-linker deconjugation in plasma over time. As the DAR of the ADC decreases during the first 1–5 days of incubation in plasma (by retro-Michael deconjugation of the maleimide moiety), the overall hydrophobicity of the ADC also decreases, leading to lower ADC clearance values. Additionally, the ingenious chemical optimizations of the deruxtecan drug-linker (self-immolative amino methylene and glycolic acid spacers directly connected to the hindered primary amine of exatecan) may largely contribute to a reduced steric hindrance and thus play a significant role in the outstanding pharmacological properties of DS-8201a [40,42].

Tra-Exa-PSAR10 and DS-8201a did not show any apparent systemic toxicity in mice at 100 mg/kg, a dose that is much greater than the dose inducing complete remissions in the HER2+xenograft models. It should be noted that the PSAR-lacking ADC Tra-Exa-PSAR0 did not induce apparent toxicity either at the same dose. At this stage and on the basis of this very exploratory toxicity study, we were not able to discriminate these three ADCs in terms of toxicity profiles or maximum tolerated doses (MTD). Comprehensive toxicological studies on PSAR-based ADCs are currently ongoing and will be reported elsewhere.

Tra-Exa-PSAR10 displayed potent anti-tumor efficacies in both breast and gastric cancer xenografts. Unsurprisingly and driven by its unfavorable PK profile, negative control ADC Tra-Exa-PSAR0 showed reduced efficacy. DS-8201a exhibited a lower anti-tumor activity than Tra-Exa-PSAR10 in the NCI-N87 gastric cancer model, despite having the same in vitro activity and PK profile. This result could be explained by the premature maleimide deconjugation of DS-8201a in plasma and by a lower bystander killing effect of the DXd payload compared to exatecan. This discrepancy in activity could also be driven by the difference on drug-linker cleavage strategy and kinetics (cathepsin sensitive quadripeptide *versus* glucuronidase-sensitive trigger) that are dependent on intracellular proteases and glucuronidases levels, respectively. In addition, the presence of extracellular glucuronidase in the tumor microenvironment reported in several studies [45,56,57] could tilt the balance in favor of Tra-Exa-PSAR10.

In the ADC field, the bystander effect is characterized by the ability of the final active metabolite to passively cross cell membranes and internalize into neighboring low-antigen-expressing cells within the targeted tumor [58]. As an example, the FDA-approved trastuzumab emtansine (comprised of the mAb trastuzumab and the anti-microtubule agent DM1 linked through a non-cleavable SMCC linker) is devoid of such a bystander effect, as the final active metabolite is the highly polar entity Lys-SMCC-DM1, which is not capable of passive membrane diffusion [59]. In contrast, the FDA-approved DS-8201a is based on a cleavable drug-linker releasing the final metabolite DXd, which is capable of passive membrane diffusion and therefore bystander killing [37]. We herein evaluated the bystander killing potency of Tra-Exa-PSAR10 and DS-8201a in a co-culture experiment of HER2+ and HER2-cells. This experiment revealed a comparable yet stronger bystander killing effect for the exatecan-based ADC. These data encourage the development of exatecan-based ADCs for the treatment of tumors with heterogeneous antigen expression, as this payload is able to passively diffuse into neighboring antigen-negative cancer cells [58,60]. We suggest that the slight difference observed in bystander effect potency between these ADCs is driven by the slight difference in passive membrane diffusion of the two closely related camptothecin derivatives, as we observed a higher permeability coefficient (Pe) for exatecan compared to DXd in a PAMPA experiment. This result may appear counterintuitive, as it could be expected that the allegedly positively charged primary amine of exatecan (not present in the DXd payload) would negatively affect passive membrane permeability. However numerous reports provide evidence that positively charged compounds can have high membrane permeabilities, and that predicting Pe of such compounds is not obvious [61,62]. Of note it is interesting to observe that the “first generation” DXd payload, which also bears a positively charged alkyl primary amine (4-aminobutanoic acid derivative of exatecan), was devoid of passive permeability capabilities [36].

Most of the ADCs that are currently approved or under clinical investigation are based on microtubule- and DNA-targeting agents. One of the main purposes of differentiated ADC payloads is to efficiently treat tumors that become resistant to these chemotherapeutic agents or resistant to the ADC per se [33,37,63,64]. To address this issue, we investigated the in vitro cytotoxicity of Tra-Exa-PSAR10 in HER2+ breast (MDA-MB-361) and esophageal (OE-19) cancer cell lines that were rendered resistant to T-DM1. The characterization of T-DM1 resistance mechanisms of these two models demonstrated no alteration of HER2 expression in resistant OE-19 cells, whereas resistant MDA-MB-361 cells demonstrated heterogeneity in HER2 expression when compared to the parental cell line [55,56]. Our results showed that Tra-Exa-PSAR10 circumvented T-DM1-resistance in both cancer models. These results suggest that exatecan-based ADCs could be clinically useful for the treatment of tumors that are resistant to maytansine-based therapies, including when the target is expressed heterogeneously.

In conclusion, we herein report an innovative highly conjugated (DAR 8) ADC based on the topoisomerase I inhibitor payload exatecan. This could be achieved while preserving favorable physicochemical and pharmacological properties of the conjugate, thanks to the use of a polysarcosine-based hydrophobicity masking entity. Tra-Exa-PSAR10 showed remarkable anti-tumor properties, outperforming the FDA-approved ADC DS-8201a in a gastric cancer model. In addition, this ADC demonstrated a strong bystander killing effect as well as potent activity in T-DM1-resistant cells, suggesting a potential broad anti-tumor activity in resistant and heterogeneous tumors.

## 4. Material and Methods

### 4.1. Drug-Linker Synthesis

Chemical preparation of drug-linkers used in the present study is described in the Supplementary Material Section of the present article.

### 4.2. Preparation of Antibody-Drug-Conjugates

A solution of trastuzumab (10 mg/mL in PBS (pH 7.4) + 1 mM EDTA—Herceptin IV from Roche) was treated with 12 molar equivalent of tris(2-carboxyethyl)phosphine (TCEP) for 2 h at 37 °C. The fully reduced antibody was buffer-exchanged with potassium phosphate 100 mM (pH 7.4) + 1 mM EDTA by 3 rounds of dilution/centrifugation using an Amicon 30K centrifugal filters device (Merck). Ten molar equivalents of drug-linker (from a 12 mM DMSO stock solution) were added to the reduced antibody, while keeping residual DMSO concentration below 10% (*v*/*v*). The solution was incubated for 30 min at room temperature. The conjugates were buffer-exchanged/purified with PBS (pH 8.0) using an Amicon 30K centrifugal filters device and were incubated at 37 °C for 24 h to promote complete hydrolysis of the succinimidyl moiety. The conjugates were buffer-exchanged again with PBS (pH 7.4) using an Amicon 30K centrifugal filters device and were sterile-filtered (0.2 µM PES filters). Final protein concentration was assessed spectrophotometrically at 280 nm using a Colibri microvolume spectrometer device (Titertek Berthold).

### 4.3. Characterization of Antibody-Drug Conjugates

Denaturing reversed phase chromatography–mass spectrometry (RPLC-MS) was performed on a Thermo UltiMate 3000 UHPLC system + Bruker Impact II Q-ToF mass spectrometer. Mobile phase A was water + 0.1% formic acid and mobile phase B was acetonitrile + 0.1% formic acid. Column was an Agilent PLRP-S 1000Å 2.1 × 150 mm 8 µm (80 °C). Gradient was 20%B to 50%B in 25 min. Flow rate was 0.4 mL/min. UV detection was monitored at 280 nm. The Q-ToF mass spectrometer was used in the *m/z* range 500–3500 (ESI+). Data were deconvoluted using the MaxEnt algorithm included in the Bruker Compass software. mAb or ADC samples were diluted with H_2_O for injection (approximately 1.5 mg/mL final ADC concentration).

Hydrophobic interaction chromatography (HIC) was performed on an Agilent 1100 HPLC system. Column was a Tosoh TSK-GEL BUTYL-NPR 4.6 × 35 mm 2.5 µm (25 °C). Mobile phase A was 1.5 M (NH_4_)_2_SO_4_ + 25 mM potassium phosphate (pH 7.0). Mobile phase B was 25 mM potassium phosphate (pH 7.0) + 15% isopropanol (*v*/*v*). Linear gradient was 0%B to 100%B in 10 min, followed by a 3 min hold at 100%B. Flow rate was 0.75 mL/min. UV detection was monitored at 220 and 280 nm.

Size exclusion chromatography (SEC) was performed on an Agilent 1100 HPLC system having an extra-column volume below 15 µL (equipped with short sections of 0.12 mm internal diameter peek tubing and a micro-volume UV flow cell). Column was an Agilent AdvanceBioSEC 300Å 4.6 × 150 mm 2.7 µm (maintained at 30 °C). Mobile phase was 100 mM sodium phosphate and 200 mM sodium chloride (pH 6.8). We added 10% acetonitrile (*v*/*v*) to the mobile phase to minimize secondary hydrophobic interactions with the stationary phase and prevent bacterial growth. Flow rate was 0.35 mL/min. UV detection was monitored at 220 and 280 nm.

### 4.4. HER2-Binding ELISA Affinity Assay

Sandwich ELISA assays were performed using 96-well high-binding ELISA plates (Corning Inc., New York, NY, USA, Cat#3590). Plates were coated using 100 µL/well of the tested monoclonal antibody or ADC in PBS (pH 7.4) at 5 µg/mL and incubated overnight at 4 °C. After 2 washes with PBS-T (PBS + 0.05% Tween-20), the plates were blocked with 200 µL/well of incubation buffer (PBS-T + 0.1% BSA) for 1 h at room temperature. The plates were washed 4 times with PBS-T, and 100 µL of a threefold dilution series of His-tagged HER2 recombinant protein (Sino Biological Inc. Cat#10004-H08H) prepared in incubation buffer was added; then, the plates were incubated for 2 h at room temperature in the dark. After 5 washes with PBS-T, plates were incubated 1 h at room temperature with 100 µL/well of HRP-conjugated anti-His Tag secondary antibody (Takara Inc. cat#631210) diluted 1:5000 in incubation buffer. After 5 washes with PBS-T, TMB substrate solution (Thermo-Fisher cat#N301) was added. Peroxidase activity was stopped with 0.18M H_2_SO_4_ and absorbance was read at 450 nm (reference wavelength 650 nm) using a Thermo Scientific MultiSkan EX microplate reader. Sigmoidal fittings were performed using GraphPad Prism 9 software.

### 4.5. Ex Vivo Plasma Stability Assays

ADC samples (>5 mg/mL solutions in PBS) were diluted with pure sterile Sprague-Dawley rat plasma (GeneTex Cat#GTX73218) in centrifuge tubes with screw cap to yield a final ADC concentration of 400 µg/mL (residual PBS volume below 10% *v*/*v*). Samples were incubated at 37 °C and aliquots were taken at time points of 5 min, 1 day, 2 days, 4 days, and 7 days (aliquots were kept frozen at −80 °C until analysis). ADCs were recovered from plasma by immunocapture using AbraMag^TM^ anti-Human magnetic beads (Eurofins Technologies Cat#544061) following the manufacturer’s protocol. Bound conjugates were extensively washed with TBS-T (TBS + 0.05% Tween-20), eluted with 0.1M glycine buffer (pH 2.0), and neutralized with 1M TBS (pH 8.0). Each sample was analyzed by denaturing reversed phase chromatography–mass spectrometry as described above.

### 4.6. Cell Culture

Human breast adenocarcinoma cell lines MDA-MB-361, MDA-MB-453, and SKBR-3 were cultured in DMEM supplemented with 10% fetal calf serum and 100 µg/mL streptomycin at 37 °C and incubated under a 5% CO_2_ atmosphere. The human breast adenocarcinoma BT-474, the gastric cancer NCI-N87, and the esophageal OE-19 cancer cell lines were cultured in RPMI medium supplemented with 10% fetal calf serum and 100 µg/mL streptomycin at 37 °C and incubated under a 5% CO_2_ atmosphere.

MDA-MB-361 and OE-19 cells resistant to T-DM1 were cultured in DMEM and RPMI complete mediums, respectively, plus 0.4 nM of T-DM1, with (TR) or without (TCR) 1 µg/mL of ciclosporin A (Cat#C3662, Sigma-Aldrich, St. Louis, MI, USA).

### 4.7. Flow Cytometry

For HER2 cell surface quantification, cells were incubated for 30 min with anti-HER2 APC-conjugated antibody (BD Bioscience, Franklin Lakes, NJ, USA, Cat#340554) or mouse IgG1k control isotype conjugated to APC (BD Pharmingen, San Diego, CA, USA, Cat#555751). For mAb or ADC cell binding, the tested compound was conjugated to APC fluorochrome using LYNX Rapid APC Antibody Conjugation Kit according to the manufacturer’s protocol (Bio-Rad, Hercules, CA, USA, Cat#LNK032APC). Analysis was performed using a BD Fortessa flow cytometer controlled by BD FACSDiva software (BD Biosciences) and data were analyzed using FlowJo software (BD Bioscience).

### 4.8. In Vitro Cytotoxicity Assays

In vitro cytotoxicity of conjugates was assessed on several antigen-positive cell lines. Cells were plated in 96-well plates at an appropriate density depending on the cell line (between 1000 and 10,000 cells per well in 100 µL of appropriate culture media) and incubated at 37 °C for 24 h. Serial dilutions of the tested compound previously dissolved in culture media were added, and incubation was carried out at 37 °C for 144 h. MTT (5 mg/mL, 20 μL, Sigma-Aldrich) was added into the wells, and incubation was continued for 2 to 4 h at 37 °C. Culture media was then carefully removed, and well content was homogeneously dissolved with 0.1 N HCl/isopropanol. Absorbance values were measured on a Thermo Scientific Multiskan EX microplate reader using a wavelength of 570 nm (with a reference wavelength of 690 nm). The IC_50_ concentration values compared to untreated control cells were determined using inhibition dose response curve fitting (GraphPad Prism 9).

### 4.9. PAMPA Permeability Assay

Passive permeabilities of exatecan mesylate and DXd (both from MedChemExpress) were compared by parallel artificial membrane permeability assay (PAMPA assay) using a GenTest^TM^ pre-coated PAMPA plate system (Corning, New York, NY, USA, Cat#353015), following the manufacturer’s protocol and calculation procedures. The assay buffer was PBS (pH 7.4) + 10% MeOH (*v*/*v*). The initial compound concentration in the donor compartment was 100 µM. Residual DMSO in the assay was kept below 1% (*v*/*v*). The permeability partitioning was realized for 5 h at room temperature while stirring the plate at 300 rpm on a Heidolph Titramax 101 device. Final concentration of tested compound in the donor and acceptor compartments was assessed by HPLC–UV, against known calibration curves of compounds.

### 4.10. In Vitro Bystander Killing Assay

SKBR-3 and A549 cells were seeded in single culture or co-culture in 96-well plates at a density of 8000 cells per well and incubated overnight at 37 °C under a 5% CO_2_ atmosphere. The incubation media was removed and 100 µL of a 10 nM solution of tested conjugate in DMEM complete medium were added to the plate and incubated for 5 days. Cells were then collected and transferred into a round-bottom 96-well plate suitable for high-throughput flow cytometry. Cells were rinsed 3 times with PBS, resuspended in 50 µL DPBS, and incubated for 30 min at room temperature in the dark with anti-HER2 APC-conjugated antibody (BD Bioscience, Cat#340554) and eBioscience Fixable Viability Dye eFluor 780 (Thermo Scientific, Waltham, MA, USA, Cat#650865-14). Flow cytometry analysis was performed using a BD Fortessa flow cytometer controlled by BD FACSDiva software (BD Biosciences), and data were analyzed using FlowJo software (BD Bioscience).

### 4.11. In Vivo Studies

All animal procedures were performed in accordance with the European Union directive 86/609/EEC. Experiments were performed under individual permit and in animal care facilities accredited by the French Ministry of Agriculture. The study was approved by the local animal ethics committee (CECCAPP). The rat PK and NCI-N87 xenograft studies were outsourced to the contract research organization (CRO) Antinéo (www.antineo.fr; accessed on 10 August 2020).

### 4.12. Rat PK Study

ADCs were injected at 3 mg/kg in female Sprague-Dawley rats (4–6 weeks old—Charles River) via the tail vein (3 animals per group, randomly assigned). Blood was drawn into citrate tubes *via* retro-orbital bleeding at 10 min, 4 h, 1 day, 2 days, 4 days, 7 days, 14 days, and 21 days; processed to plasma; and stored at −80 °C until analysis. ADC concentration was assessed using a human IgG ELISA kit (Stemcell Technologies, Cambridge, MA, USA, Cat#01994) according to the manufacturer’s protocol. Standard curves of trastuzumab antibody were used for quantification. Pharmacokinetic parameters (clearance, half-life, and AUC) were calculated by two-compartmental analysis using Microsoft Excel software incorporating PK functions (add-in developed by Usansky et al., Department of Pharmacokinetics and Drug Metabolism, Allergan, Irvine, CA, USA).

### 4.13. In Vivo Efficacy Experiments

In vivo efficacy studies were conducted in severe combined immunodeficiency (SCID) mice (*n* = 6). We suspended 5 × 10^6^ BT-474 or NCI-N87 cells in 0.2 mL of PBS (pH 7.4) and injected them subcutaneously in the left flank of mice. For the breast cancer BT-474 xenograft experiment, treatment was initiated when the tumor volume averaged 150 mm^3^ and was administered once intravenously at an ADC concentration of 10 mg/kg (*n* = 6 mice per group). For the gastric cancer NCI-N87 xenograft experiment, treatment was initiated when the tumor volume averaged 150 mm^3^ and was administered once intravenously at a sub-curative dose of 1 mg/kg (*n* = 8 mice per group). Tumor volumes were measured every 3–5 days using a caliper device (length x width) and calculated using the following formula V = 4/3 × π × R^3^, where R represents the radius. Mice were sacrificed when the tumor volume exceeded 1500 mm^3^. No significant body weight changes were observed during these studies.

### 4.14. Mice Tolerability Experiments

To assess mouse tolerability of ADCs of the present study, we treated SCID mice (*n* = 3) with a single intraperitoneal dose of 100 mg/kg of ADC compound. Mice were observed for weight loss or apparent signs of toxicity over the course of 10 days.

### 4.15. Statistics

In vitro experiments were conducted at least 3 times, and data are presented as means +/− SD or single representative experiments. Statistical significance was evaluated using Student’s *t*-test and calculated using GraphPad Prism 9 software. IC_50_’s and EC_50_’s were calculated using GraphPad Prism 9 software. In vivo experiment data are represented as means +/− SD. Statistical significance was evaluated using Mann–Whitney test and calculated using GraphPad Prism 9 software. *p*-values are represented as * (*p* < 0.05), ** (*p* < 0.01), and *** (*p* < 0.001), and “ns” stands for non-significant.

## Data Availability

Not applicable.

## Supplementary Materials

Supplementary figures and organic syntheses. The following are available online at https://www.mdpi.com/1424-8247/14/3/247/s1, Figure S1: Chemical structures of Exatecan and DXd payloads, Figure S2: Structures of trastuzumab-based antibody-drug conjugates, Figure S3: Representative RPLC-QToF characterization of ADCs, Figure S4: Pharmacokinetic parameters (Sprague-Dawley rat PK study), Figure S5: Survival curve of the SCID/NCI-N87 xenograft study.
