# Supplementary material for: Exatecan Antibody Drug Conjugates Based on a Hydrophilic Polysarcosine Drug-Linker Platform
Source: Pharmaceuticals (Basel). 2021 Mar 9;14(3):247. doi: 10.3390/ph14030247 (PMC8000490; doi:10.3390/ph14030247)
Supplement: Supplementary file 1 [file pharmaceuticals-14-00247-s001.pdf]

Supplementary materials

Exatecan Antibody Drug Conjugates Based on a Hydrophilic Polysarcosine Drug-Linker Platform

Louise Conilh [1,2], Guy Fournet [3], Eric Fourmaux [1,2], Angélique Murcia [1,3], Eva-Laure Matera [2]
Benoît Joseph [3,†], Charles Dumontet [2,4,†], Warren Viricel [1,†]

* These authors contributed equally

Affiliations

[1] Mablink Bioscience, 69 rue de la république, 69002 Lyon, France

[2] Centre de Recherche en Cancérologie de Lyon, INSERM 1052, CNRS 5286, Université de Lyon, 69008 Lyon, France

[3] Institut de Chimie et Biochimie Moléculaires et Supramoléculaires, UMR CNRS 5246, Université de Lyon, 69100 Villeurbanne, France

[4] Hospices Civils de Lyon, 69000 Lyon, France

Corresponding author

Warren Viricel: w.viricel@mablink.com

Supplementary figures

Figure S1. Chemical structures of Exatecan and DXd payloads

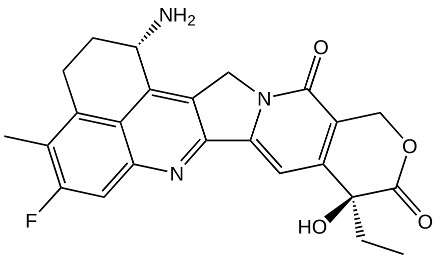

Exatecan (active payload of drug-linkers Tra-Exa-PSAR10 & Tra-Exa-PSAR0)

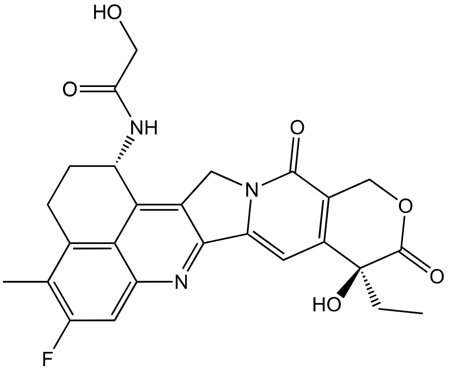

DXd (active payload of drug-linker deruxtecan)

Figure S2. Structures of trastuzumab-based antibody-drug conjugates (ADC)

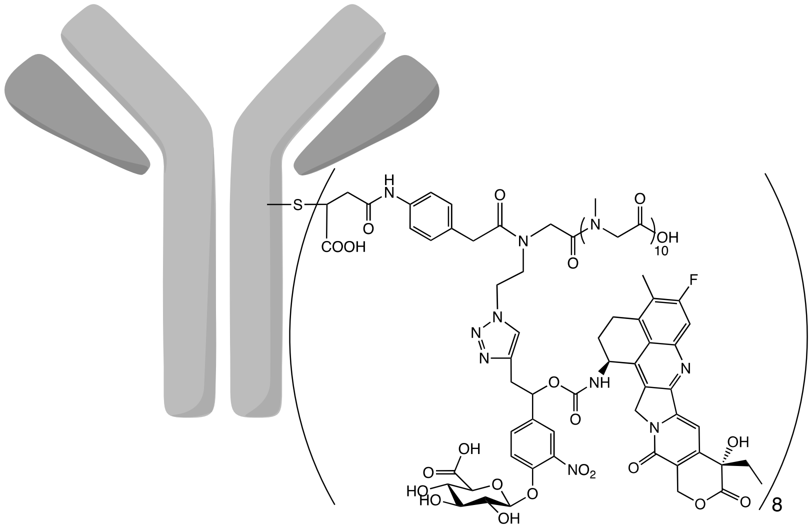

Tra-Exa-PSAR10

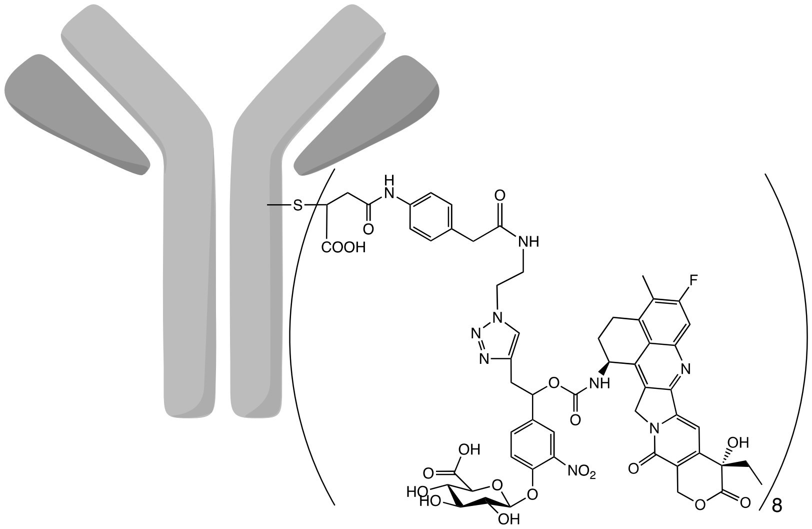

Tra-Exa-PSAR0

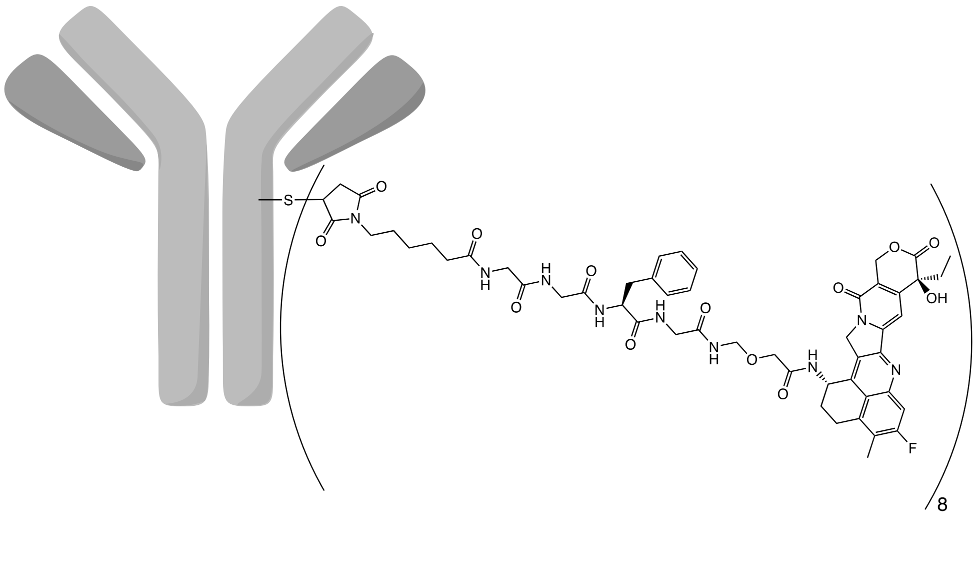

Tra-deruxtecan

Figure S3. Representative RPLC-QToF characterization of ADCs

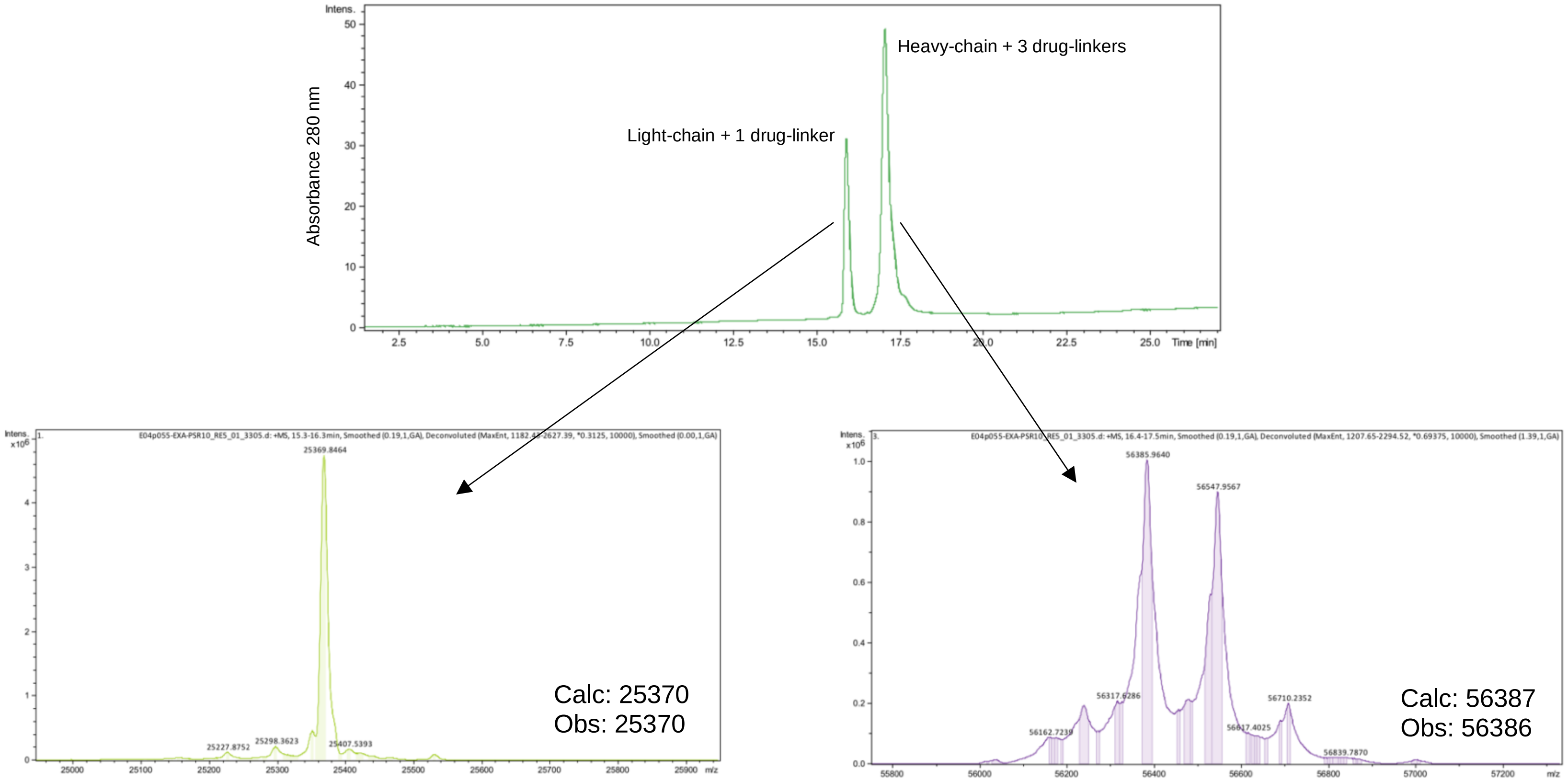

Deconvoluted LC-1d

Deconvoluted HC-3d

Reverse phase liquid chromatography mass spectrometry (RPLC-QToF) profile of ADC Tra-Exa-PSAR10 showed for illustration purposes. Conjugates exhibited one light chain with 1 drug-linker attached (LC-1d) and one heavy chain with 3 drug-linkers attached (HC-3d) absorbance peaks on their denaturing RPLC chromatogram (DAR8 conjugates).

- Tra-Exa-PSAR10 (DAR8)
    - Deconvoluted LC-1d Calc: 25370 ; Obs: 25370 / Deconvoluted HC-3d Calc: 56387 ; Obs: 56386 (major glycoform G0F)
    - Monomeric purity : 95%+
    - HIC retention time : 5.1 min
- Tra-Exa-PSAR0 (DAR8)
    - Deconvoluted LC-1d Calc: 24602 ; Obs: 24601 / Deconvoluted HC-3d Calc: 54081 ; Obs: 54079 (major glycoform G0F)
    - Monomeric purity : 95%+
    - HIC retention time : 5.4 min
- Tra-deruxtecan (DAR8)
    - Deconvoluted LC-1d Calc: 24474 ; Obs: 24473 / Deconvoluted HC-3d Calc: 53698 ; Obs: 53696 (major glycoform G0F)
    - Monomeric purity : 95%+
    - HIC retention time : 6.5 min

Figure S4. Pharmacokinetic parameters (Sprague-Dawley rat PK study)

	TRASTUZUMAB	TRA-EXA-PSAR10	TRA-EXA-PSAR0	TRA-DERUXTECAN
Clearance (mL/day/kg)	47.3	48.6	134.9	44.8
$AUC_{0\text{-}inf}$ (day x µM)	243.8	215.6	77.6	233.6
Half-life (days)	13.5	8.8	3.2	8.9

Figure S5. Survival curve of the SCID/NCI-N87 xenograft study

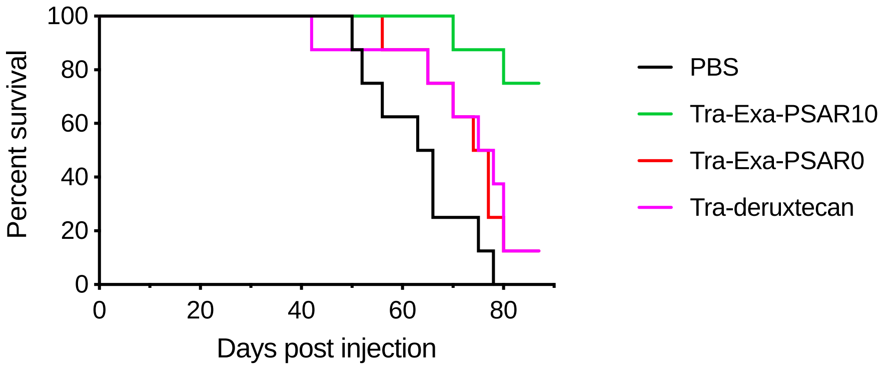

Survival curves of the SCID/NCI-N87 xenograft study. ADCs were injected once intravenously at a sub-curative dose of 1mg/kg. No body-weight changes were observed during the study.

Supplementary experimental procedures

Chemical reagents and general methods

All solvents and reagents were obtained from reputable commercial sources (Sigma-Aldrich, Fluorochem, Acros Organics, Alfa Aesar, Thermo Fisher, Carbosynth) and used without further purification unless stated otherwise. Anhydrous solvents were purchased from Sigma-Aldrich. Fmoc-aminoacids and 2-chlorotrityl 1% DVB 100-200 mesh resin (pre-loaded with first Fmoc-sarcosine aminoacid) were purchased from Christof Senn Laboratories. Exatecan Mesylate (CAS#169869-90-3), DXd (CAS#1599440-33-1) and deruxtecan drug-linker (CAS#1599440-13-7) were purchased from MedChemExpress. Trastuzumab (Herceptin® IV) was purchased from Roche.

On-resin synthesis was performed in empty SPE plastic tubes equipped with a 20µm polyethylene frit (Sigma-Aldrich). A Titramax 101 platform shaker (Heidolph) was used for agitation. Unless stated otherwise, all chemical reactions were carried out at room temperature under an inert argon atmosphere.

Mass spectroscopy analysis was performed by the Centre Commun de Spectrométrie de Masse (CCSM) of the Institut de Chimie et Biochimie Moléculaires et Supramoléculaires - UMR CNRS 5246.

Normal phase flash chromatography was performed on Teledyne Isco CombiFlash® Rf200 devices using Macherey-Nagel Chromabond® flash cartridges (40-63µm). Reverse phase chromatography was performed using Biotage® Sfär C18 Duo 100Å 30µm cartridges or Interchim PuriFlash RP-AQ (30µm) cartridges on Teledyne Isco Combiflash® Rf200 devices or using an Agilent 1100 preparative HPLC system.

Chemical reactions and compound characterization were respectively monitored and analyzed by thin-layer chromatography using pre-coated 40-63µm silica gel (Macherey-Nagel), HPLC-UV (Agilent 1100 systems) or UHPLC-UV/MS (Thermo UltiMate 3000 UHPLC system equipped with a Bruker Impact II™ Q-ToF mass spectrometer or Agilent 1260 HPLC system equipped with a Bruker MicrOTOF-QII mass spectrometer).

HPLC methods

HPLC Method 1: Agilent 1100 HPLC system equipped with DAD detection. Mobile phase A was water + 0.1% TFA and mobile phase B was acetonitrile. Column was an Agilent Zorbax SB-Aq 4.6x150mm 5µm (room temperature). Linear gradient was 0%B to 50%B in 30 min, followed by a 5 min hold at 50%B. Flow rate was 1.0 mL/min.

HPLC Method 2: Agilent 1100 HPLC system equipped with DAD detection. Mobile phase A was water + 0.1% TFA and mobile phase B was acetonitrile. Column was an Agilent Poroshell 120 EC-C18 3.0x50mm 2.7µm (room temperature). Linear gradient was 5%B to 80%B in 9 min, followed by a 1 min hold at 80%B. Flow rate was 0.8 mL/min.

HPLC Method 3: Agilent 1100 HPLC system equipped with DAD detection. Mobile phase A was water + 0.1% TFA and mobile phase B was acetonitrile. Column was an Agilent Poroshell 120 EC-C18 3.0x50mm 2.7µm (room temperature). Linear gradient was 5%B to 80%B in 20 min, followed by a 2 min hold at 80%B. Flow rate was 0.8 mL/min.

HPLC Method 4 (preparative method): Agilent 1100 preparative binary HPLC system equipped with dual-loop auto-injector, DAD detection and fraction collector. Mobile phase A was water + 0.1% TFA and mobile phase B was acetonitrile. Column was a Waters SunFire C18 OBD Prep Column, 100Å, 5 µm, 19mm x 250mm (room temperature). Linear gradient was 10%B to 60%B in 40 min, followed by a 5 min hold at 60%B. Flow rate was 25 mL/min.

Organic synthesis procedures

1. Synthesis of polysarcosine intermediate NH-N_3-PSAR10

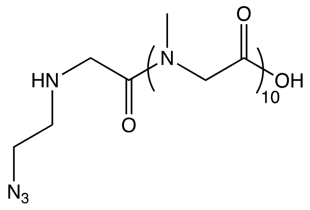

Monodisperse polysarcosine intermediate NH-N_3-PSAR10 was synthesized on 2-chlorotrityl solid-support using submonomer synthesis procedures[1], as already described in Viricel et al.[2] and in patent number WO2019081455.

Final compound NH-N_3-PSAR10 was obtained as an off-white solid (198.8 mg / 27% yield based on initial resin loading). MS (ESI+): $[M+H]^+$ = 855.4. HPLC Method 1 retention time = 11.3 min.

2. Synthesis of intermediate compound alkyne-glucuronide-Exatecan

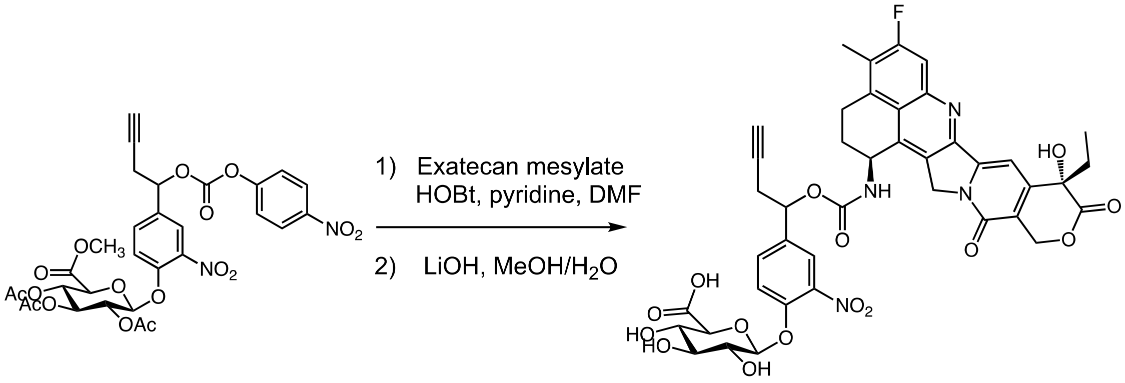

Compound alkyne-glucuronide-Exatecan

132.1 mg (0.192 mmol) of starting material (synthesized as described in Renoux et al.)[3], 102 mg (0.192 mmol) of Exatecan mesylate and 26 mg (0.192 mmol) of HOBt were dissolved with 1 mL of a 8:2 (v/v) mixture of anhydrous DMF/pyridine. The reaction was stirred 16 hours at room temperature and volatiles were evaporated under reduced pressure. The crude residue was purified by chromatography on silica gel (DCM/MeOH gradient from 98:2 to 90:10) to afford 165 mg (87%) of intermediate compound (yellow solid) that was directly engaged into the deprotection step. ESI^+ $[M+H]^+$ = 985.3.

165 mg (0.168 mmol) of this compound was dissolved in MeOH/THF 1:1 v/v (16 mL) at 0°C. LiOH monohydrate (70.3 mg / 1.675 mmol) was dissolved in water (1.6 mL) and was slowly added to the reaction vessel. After stirring at 0°C for 70 min, the mixture was neutralized with

acetic acid (131 mg / 2.18 mmol) and concentrated under reduced pressure. The resulting material was taken up in a water/MeOH/DMF solution (1:1:1 v/v) and purified on a Biotage® Sfär C18 Duo 100Å 30µm cartridge. Mobile phase A was water + 0.05% TFA and mobile phase B was acetonitrile + 0.05% TFA. The gradient ranged from 10 to 50% B.

Compound alkyne-glucuronide-Exatecan was obtained as a yellow solid (98 mg / 69%). LC-HRMS *m/z* (ESI^+): Calc $[M+H]^+$ = 845.2312 ; Exp $[M+H]^+$ = 845.2360 ; Error = -4.8 ppm. HPLC Method 3 retention time = 8.0 min.

3. Synthesis of final drug-linker Exa-PSAR10

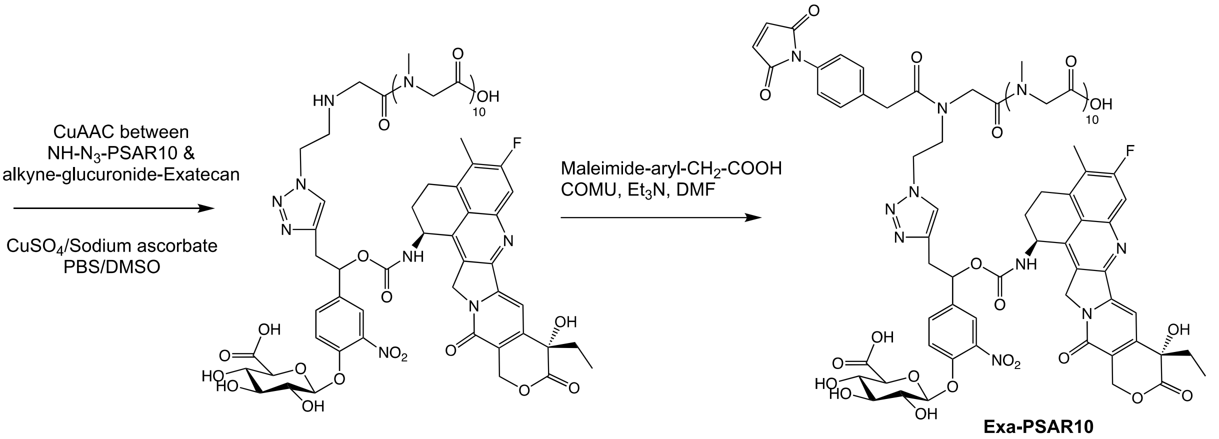

30.0 mg (0.036 mmol) of alkyne-glucuronide-Exatecan and 42.5 mg (0.049 mmol) of NH-N_3-PSAR10 were dissolved in a 1:1 (v/v) solution of 100 mM PBS (pH = 7.5) and DMSO, in order to reach a 0.060M concentration of compound alkyne-glucuronide-Exatecan. Freshly prepared $CuSO_4$ pentahydrate and sodium ascorbate solutions (approximately 250 mg/mL) were then sequentially added into the reaction vial in order to reach 0.08 molar equivalent Cu and 1 molar equivalent of sodium ascorbate (based on compound alkyne-glucuronide-Exatecan molar equivalent in the reaction mixture). The reaction was purged with argon, stirred at room temperature for 1 hour and diluted with a 0.1% TFA solution in water/acetonitrile 2:1 (v/v). Purification using preparative HPLC Method 4 as described above yielded 41 mg (68%) of intermediate compound as a yellow solid that was directly engaged in the next reaction step.

7.8 mg (0.034 mmol) of commercial 2-(4-(2,5-dioxo-2*H*-pyrrol-1(5*H*)-yl)phenyl)acetic acid (CAS#91574-45-7) and 13.4 mg (0.031 mmol) of COMU were dissolved in anhydrous DMF (0.1M concentration of maleimide compound). 8.5 mg (0.084 mmol) of Et_3N was added. The reaction was pre-incubated 2 minutes at room temperature and transferred onto 41 mg (0.024 mmol)

of previous compound (pre-weighted in a reaction vial). The reaction was stirred for 30 min until entire conversion of the reaction was observed by HPLC. The reaction mixture was then diluted with a 1% TFA solution in water/acetonitrile 1:1 (v/v) and purified using preparative HPLC Method 4 to afford 14.9 mg (33%) of final drug-linker compound Exa-PSAR10 as a yellow solid. HRMS *m/z* (ESI$^+$): Calc $[M+2H]^{2+}$ = 956.8585 ; Exp $[M+2H]^{2+}$ = 956.8571 ; Error = 1.4 ppm. HPLC Method 3 retention time = 8.65 and 8.76 min (equimolar diastereoisomeric mixture).

4. Synthesis of final drug-linker Exa-PSAR0

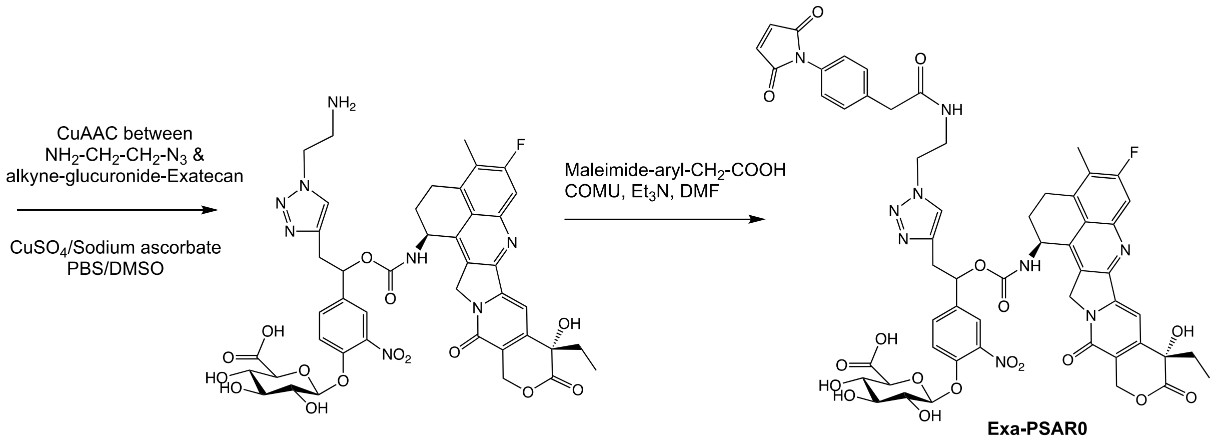

Intermediate compound was synthesized starting from alkyne-glucuronide-Exatecan and 2-azidoethan-1-amine, following the CuAAC procedure described above. 6.2 mg (25% yield) of intermediate compound was obtained as a yellow solid and was directly engaged in the next reaction step.

2-(4-(2,5-dioxo-2*H*-pyrrol-1(5*H*)-yl)phenyl)acetic acid coupling was realized as described above, using COMU as coupling reagent. The reaction mixture was purified using preparative HPLC Method 4 to afford 4.2 mg (53%) of final drug-linker compound Exa-PSAR0 as a yellow solid. HRMS *m/z* (ESI$^+$): Calc $[M+H]^+$ = 1144.3331 ; Exp $[M+H]^+$ = 1144.3351 ; Error = -1.8 ppm. HPLC Method 3 retention time = 9.6 min (equimolar diastereoisomeric mixture).

References

(1) Zuckermann, R. N.; Kerr, J. M.; Kent, S. B. H.; Moos, W. H. Efficient Method for the Preparation of Peptoids [Oligo(N-Substituted Glycines)] by Submonomer Solid-Phase Synthesis. *J. Am. Chem. Soc.* **1992**, *114* (26), 10646–10647. https://doi.org/10.1021/ja00052a076.

(2) Viricel, W.; Fournet, G.; Beaumel, S.; Perrial, E.; Papot, S.; Dumontet, C.; Joseph, B. Monodisperse Polysarcosine-Based Highly-Loaded Antibody-Drug Conjugates. *Chem. Sci.* **2019**, *10*, 4048-4053. https://doi.org/10.1039/C9SC00285E.

(3) Renoux, B.; Raes, F.; Legigan, T.; Péraudeau, E.; Eddhif, B.; Poinot, P.; Tranoy-Opalinski, I.; Alsarraf, J.; Koniev, O.; Kolodych, S.; Lerondel, S.; Pape, A. L.; Clarhaut, J.; Papot, S. Targeting the Tumour Microenvironment with an Enzyme-Responsive Drug Delivery System for the Efficient Therapy of Breast and Pancreatic Cancers. *Chem. Sci.* **2017**, *8*, 3427–3433. https://doi.org/10.1039/C7SC00472A.
